# *N*-[4-(Ethyl­sulfamo­yl)phen­yl]acetamide

**DOI:** 10.1107/S1600536811033472

**Published:** 2011-08-27

**Authors:** Jeveria Rehman, Ejaz, Islam Ullah Khan, William T. A. Harrison, Sidra Farid

**Affiliations:** a Materials Chemistry Laboratry, Department of Chemistry, GC University, Lahore 54000, Pakistan; b Department of Chemistry, University of Aberdeen, Meston Walk, Aberdeen AB24 3UE, Scotland

## Abstract

The title compound, C_10_H_14_N_2_O_3_S, crystallized with two mol­ecules (*A* and *B*) in the asymmetric unit. The terminal methyl group of the ethyl­sulfonamide moiety in mol­ecule *B* is disordered over two sets of sites with an occupancy ratio of 0.61 (1):0.39 (1). Both mol­ecules have L-shaped conformations. In mol­ecule *A*, the dihedral angles between the benzene ring and its ethyl­sulfonamide and methyl­amide substituents are 83.5 (3) and 13.34 (18)°, respectively. Equivalent values for mol­ecule *B* are 87.9 (3) and 6.32 (16)°, respectively. The C—S—N—C torsion angles are 66.5 (3)° for A and −64.4 (3)° for *B*, indicating similar twists about the S—N bonds, but in opposite senses. In the crystal, the *A* mol­ecules are linked by pairs of N_s_—H⋯O (s = sulfonamide) hydrogen bonds, generating inversion dimers containing *R*_2_^2^(8) rings, while the *B* mol­ecules are linked by N_s_—H⋯O hydrogen bonds into *C*(10) [100] chains. Finally, N_a_—H⋯O (a = amide) hydrogen bonds link the *A-*mol­ecule dimers and *B*-mol­ecule chains into a three-dimensional network.

## Related literature

For related structures, see: Hou *et al.* (2009 ▶); Khan *et al.* (2011 ▶); Rehman *et al.* (2011 ▶).
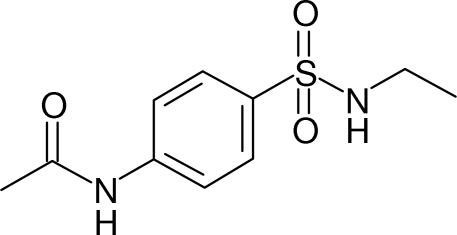


## Experimental

### 

#### Crystal data

C_10_H_14_N_2_O_3_S
*M* *_r_* = 242.29
Triclinic, 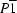

*a* = 8.2766 (3) Å
*b* = 12.1728 (4) Å
*c* = 13.5041 (4) Å
α = 70.130 (2)°
β = 73.935 (2)°
γ = 71.517 (2)°
*V* = 1191.56 (7) Å^3^
*Z* = 4
Mo *K*α radiation
μ = 0.27 mm^−1^
*T* = 296 K
0.40 × 0.35 × 0.20 mm

#### Data collection

Bruker APEXII CCD diffractometer
Absorption correction: multi-scan (*SADABS*; Bruker, 2001 ▶) *T*_min_ = 0.901, *T*_max_ = 0.949
18017 measured reflections
4310 independent reflections
2701 reflections with *I* > 2σ(*I*)
*R*_int_ = 0.049

#### Refinement

*R*[*F* ^2^ > 2σ(*F* ^2^)] = 0.046
*wR*(*F* ^2^) = 0.117
*S* = 1.04
4310 reflections
317 parameters
4 restraints
H atoms treated by a mixture of independent and constrained refinement
Δρ_max_ = 0.23 e Å^−3^
Δρ_min_ = −0.29 e Å^−3^

### 

Data collection: *APEX2* (Bruker, 2007 ▶); cell refinement: *SAINT* (Bruker, 2007 ▶); data reduction: *SAINT*; program(s) used to solve structure: *SHELXS97* (Sheldrick, 2008 ▶); program(s) used to refine structure: *SHELXL97* (Sheldrick, 2008 ▶); molecular graphics: *ORTEP-3* (Farrugia, 1997 ▶); software used to prepare material for publication: *SHELXL97*.

## Supplementary Material

Crystal structure: contains datablock(s) I, global. DOI: 10.1107/S1600536811033472/su2301sup1.cif

Structure factors: contains datablock(s) I. DOI: 10.1107/S1600536811033472/su2301Isup2.hkl

Additional supplementary materials:  crystallographic information; 3D view; checkCIF report

## Figures and Tables

**Table 1:** Hydrogen-bond geometry (Å, °)

*D*—H⋯*A*	*D*—H	H⋯*A*	*D*⋯*A*	*D*—H⋯*A*
N1—H1*N*⋯O2^i^	0.79 (3)	2.13 (3)	2.914 (3)	173 (3)
N2—H2*N*⋯O4^ii^	0.80 (2)	2.21 (2)	3.006 (3)	169 (3)
N3—H3*N*⋯O6^iii^	0.83 (3)	2.03 (3)	2.854 (3)	173 (3)
N4—H4*N*⋯O3^iv^	0.75 (2)	2.21 (2)	2.960 (3)	174 (3)

Symmetry codes: (i) 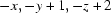; (ii) 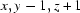; (iii) 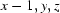; (iv) 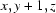.

**Fig. 1.**
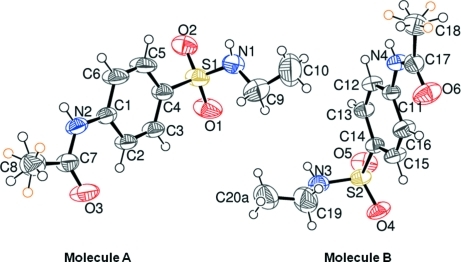
The molecular structure of the two independent molecules (A and B) of the title compound, showing the numbering scheme and 50% displacement ellipsoids. Only the major disordered component (C20A) for atom C20 is shown. The disordered methyl H-atom sites for C8 and C18 are shown in black and orange.

**Fig. 2.**
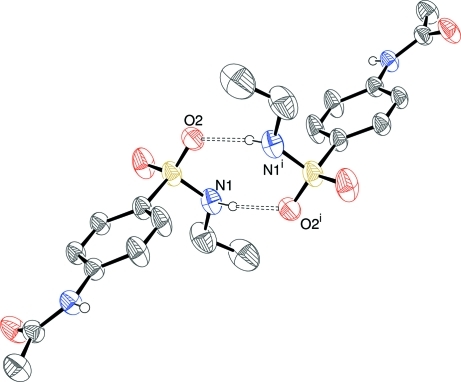
An R22(8) inversion dimer of A molecules in the crystal of the title compound, linked by pairs of N—H···O hydrogen bonds [Symmetry code: (i) -x, -y+1, -z+2; C-bound H atoms have been omitted for clarity].

**Fig. 3.**
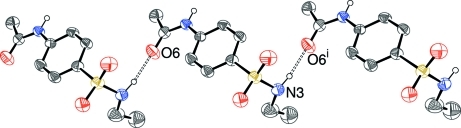
A fragment of a C(10) chain of B molecules in the crystal of the title compound, linked by N—H···O hydrogen bonds [Symmetry code: (i) x-1, y, z; C-bound H atoms have been omitted for clarity].

## supplementary crystallographic information

### Comment

As part of our ongoing structural studies of sulfonamides (Khan *et al.*, 2011; Rehman *et al.*, 2011), the synthesis and crystal structure of the title compound are reported on herein. The related stucture of *N*-(*p*-acetamidobenzenesulfonyl)glycine has been described previously (Hou *et al.*, 2009).

The title compound, C_10_H_14_N_2_O_3_S, crystallized with two molecules (A and B) in the asymmetric unit (Fig. 1). The -CH_3_ group of the ethylsulfonamide moiety (atom C20) in molecule B is disordered over two positions [C20a and C20b with occupancies 0.61 (1):0.39 (1)]. Both molecules have *L*-shaped conformations in which the ethylsulfonamide group is roughly perpendicular to the benzene ring, but the methyl-amide group is almost coplanar with the same ring. In molecule A the dihedral angles between the benzene ring (C1-C6) and the ethylsulfonamide (S1,N1,C9,C10) and methylamide (N2,C7,O3,C8) moieties are 83.5 (3) and 13.34 (18)°, respectively. The equivalent values for molecule B [benzene ring (C11-C16); ethylsulfonamide (S2,N3,C19,C20a); methylamide (N4,C17,O6,C18)] are 87.9 (3) and 6.32 (16)°, respectively. The C—S—N—C torsion angles are 66.5 (3)° for A and -64.4 (3)° for B, indicating similar twists about the S—N bonds in the two molecules, but in opposite senses. Similar twists about the equivalent S—N bonds were seen in 4-methyl-*N*-(4-aminophenyl)benzenesulfonamide (Rehman *et al.*, 2011).

In the crystal, the A molecules are linked by pairs of N_s_—H···O (s = sulfonamide) hydrogen bonds to generate inversion dimers containing *R*^2^_2_(8) rings (Fig. 2), while the B molecules are linked by N_s_—H···O hydrogen bonds into C(10) [100] chains (Fig. 3). Finally, N_a_—H···O (a = amide) hydrogen bonds link the dimers and chains into a three-dimensional network - see Table 1 for details of the hydrogen bonding.

### Experimental

Ethyl amine (1 mmol, 0.0654 ml) was dissolved in distilled water (20 ml) in a round bottom flask (100 ml) and 4-(acetylamino)benzenesulfonyl chloride (1 mmol, 0.23367 g) was added with stirring at room temperature while keeping the pH of solution between 8.0–9.0 with sodium carbonate solution (3%). After 4 h, the white precipitate formed was filtered, washed with distilled water and dried. Colourless block-like crystals of the title compound were grown from methanol by slow evaporation.

### Refinement

Atom C20 and its attached H atoms were modelled as being disordered over two sets of sites with occupancies 0.61 (1):0.39 (1). The N-bound H atoms were located in difference Fourier maps and their positions were freely refined with the constraint *U*_iso_(H) = 1.2*U*_eq_(N) applied. The C-bound hydrogen atoms were placed in calculated positions (C—H = 0.93–0.97 Å) and refined as riding atoms with *U*_iso_(H) = k × *U*_eq_(C), where k = 1.5 for methyl H-atoms and k = 1.2 for all other H-atoms. The methyl groups were allowed to rotate, but not to tip, to best fit the electron density. The methyl H-atoms attached to C8 and C18 were modelled as being equally disordered over two sets of sites, with occupancies 0.5:0.5.

### Crystal data

**Table:**

C_10_H_14_N_2_O_3_S	*Z* = 4
*M**_r_* = 242.29	*F*(000) = 512
Triclinic, *P*1	*D*_x_ = 1.351 Mg m^−^^3^
Hall symbol: -P 1	Mo *K*α radiation, λ = 0.71073 Å
*a* = 8.2766 (3) Å	Cell parameters from 2513 reflections
*b* = 12.1728 (4) Å	θ = 2.6–23.2°
*c* = 13.5041 (4) Å	µ = 0.27 mm^−^^1^
α = 70.130 (2)°	*T* = 296 K
β = 73.935 (2)°	Block, colourless
γ = 71.517 (2)°	0.40 × 0.35 × 0.20 mm
*V* = 1191.56 (7) Å^3^	

### Data collection

**Table:**

Bruker APEXII CCD diffractometer	4310 independent reflections
Radiation source: fine-focus sealed tube	2701 reflections with *I* > 2σ(*I*)
graphite	*R*_int_ = 0.049
ω scans	θ_max_ = 25.3°, θ_min_ = 1.6°
Absorption correction: multi-scan (*SADABS*; Bruker, 2001)	*h* = −9→9
*T*_min_ = 0.901, *T*_max_ = 0.949	*k* = −14→14
18017 measured reflections	*l* = −16→16

### Refinement

**Table:**

Refinement on *F*^2^	Primary atom site location: structure-invariant direct methods
Least-squares matrix: full	Secondary atom site location: difference Fourier map
*R*[*F*^2^ > 2σ(*F*^2^)] = 0.046	Hydrogen site location: inferred from neighbouring sites
*wR*(*F*^2^) = 0.117	H atoms treated by a mixture of independent and constrained refinement
*S* = 1.04	*w* = 1/[σ^2^(*F*_o_^2^) + (0.0542*P*)^2^] where *P* = (*F*_o_^2^ + 2*F*_c_^2^)/3
4310 reflections	(Δ/σ)_max_ = 0.001
317 parameters	Δρ_max_ = 0.23 e Å^−^^3^
4 restraints	Δρ_min_ = −0.29 e Å^−^^3^

### Special details

**Table:**

Geometry. All esds (except the esd in the dihedral angle between two l.s. planes) are estimated using the full covariance matrix. The cell esds are taken into account individually in the estimation of esds in distances, angles and torsion angles; correlations between esds in cell parameters are only used when they are defined by crystal symmetry. An approximate (isotropic) treatment of cell esds is used for estimating esds involving l.s. planes.
Refinement. Refinement of F^2^ against ALL reflections. The weighted R-factor wR and goodness of fit S are based on F^2^, conventional R-factors R are based on F, with F set to zero for negative F^2^. The threshold expression of F^2^ > 2sigma(F^2^) is used only for calculating R-factors(gt) etc. and is not relevant to the choice of reflections for refinement. R-factors based on F^2^ are statistically about twice as large as those based on F, and R- factors based on ALL data will be even larger.

### Fractional atomic coordinates and isotropic or equivalent isotropic displacement parameters (Å^2^)

**Table:**

	*x*	*y*	*z*	*U*_iso_*/*U*_eq_	Occ. (<1)
C1	0.2008 (3)	0.0487 (2)	0.95681 (17)	0.0464 (6)	
C2	0.1609 (3)	0.1059 (2)	0.85534 (17)	0.0505 (7)	
H2	0.1827	0.0615	0.8069	0.061*	
C3	0.0899 (4)	0.2270 (2)	0.82678 (18)	0.0525 (7)	
H3	0.0648	0.2649	0.7584	0.063*	
C4	0.0548 (4)	0.2939 (2)	0.89748 (18)	0.0520 (7)	
C5	0.0892 (5)	0.2362 (3)	1.0001 (2)	0.0727 (10)	
H5	0.0637	0.2801	1.0494	0.087*	
C6	0.1599 (4)	0.1162 (3)	1.02838 (19)	0.0704 (9)	
H6	0.1816	0.0782	1.0976	0.084*	
C7	0.3582 (4)	−0.1539 (2)	0.9343 (2)	0.0515 (7)	
C8	0.4335 (4)	−0.2783 (3)	0.9973 (2)	0.0711 (9)	
H8A	0.4104	−0.2813	1.0717	0.107*	0.50
H8B	0.5566	−0.2995	0.9720	0.107*	0.50
H8C	0.3820	−0.3341	0.9887	0.107*	0.50
H8D	0.4889	−0.3286	0.9499	0.107*	0.50
H8E	0.3428	−0.3104	1.0496	0.107*	0.50
H8F	0.5173	−0.2758	1.0329	0.107*	0.50
C9	0.2775 (5)	0.5052 (4)	0.7663 (3)	0.1020 (13)	
H9A	0.2468	0.5355	0.6958	0.122*	
H9B	0.3408	0.4214	0.7761	0.122*	
C10	0.3891 (6)	0.5758 (4)	0.7731 (3)	0.1241 (16)	
H10A	0.3247	0.6580	0.7662	0.186*	
H10B	0.4890	0.5726	0.7164	0.186*	
H10C	0.4257	0.5423	0.8410	0.186*	
S1	−0.02930 (11)	0.45083 (7)	0.85864 (5)	0.0632 (3)	
O1	−0.0688 (3)	0.48292 (18)	0.75523 (15)	0.0899 (8)	
O2	−0.1616 (3)	0.48336 (18)	0.94495 (15)	0.0701 (6)	
O3	0.3705 (3)	−0.12737 (18)	0.83768 (13)	0.0745 (6)	
N1	0.1197 (4)	0.5143 (2)	0.8485 (2)	0.0677 (8)	
H1N	0.132 (4)	0.509 (3)	0.906 (2)	0.084 (12)*	
N2	0.2774 (3)	−0.0745 (2)	0.99202 (17)	0.0509 (6)	
H2N	0.273 (3)	−0.098 (2)	1.0556 (19)	0.057 (8)*	
C11	0.5045 (3)	0.8474 (2)	0.55157 (16)	0.0400 (6)	
C12	0.3254 (3)	0.8783 (2)	0.57472 (17)	0.0464 (7)	
H12	0.2680	0.8955	0.6394	0.056*	
C13	0.2316 (3)	0.8836 (2)	0.50377 (17)	0.0462 (6)	
H13	0.1110	0.9035	0.5205	0.055*	
C14	0.3162 (3)	0.8595 (2)	0.40685 (16)	0.0429 (6)	
C15	0.4946 (4)	0.8336 (2)	0.38109 (18)	0.0530 (7)	
H15	0.5516	0.8194	0.3152	0.064*	
C16	0.5891 (4)	0.8287 (2)	0.45299 (18)	0.0542 (7)	
H16	0.7095	0.8127	0.4350	0.065*	
C17	0.7625 (4)	0.8011 (2)	0.63274 (19)	0.0471 (6)	
C18	0.8106 (4)	0.7976 (3)	0.7326 (2)	0.0613 (8)	
H18A	0.7076	0.8236	0.7814	0.092*	0.50
H18B	0.8859	0.8502	0.7151	0.092*	0.50
H18C	0.8694	0.7167	0.7655	0.092*	0.50
H18D	0.9343	0.7701	0.7266	0.092*	0.50
H18E	0.7560	0.7435	0.7929	0.092*	0.50
H18F	0.7725	0.8769	0.7425	0.092*	0.50
C19	0.2575 (5)	0.6154 (3)	0.3897 (3)	0.0936 (11)	
H19A	0.3533	0.6220	0.4139	0.112*	0.61
H19B	0.3027	0.6012	0.3196	0.112*	0.61
H19C	0.2166	0.5623	0.3680	0.112*	0.39
H19D	0.3637	0.6287	0.3398	0.112*	0.39
C20A	0.1990 (9)	0.5089 (5)	0.4637 (5)	0.109 (2)	0.61
H20A	0.2979	0.4448	0.4832	0.131*	0.61
H20B	0.1359	0.4833	0.4293	0.131*	0.61
H20C	0.1251	0.5289	0.5270	0.131*	0.61
C20B	0.2956 (10)	0.5581 (9)	0.4878 (7)	0.097 (3)	0.39
H20D	0.3904	0.4885	0.4843	0.116*	0.39
H20E	0.1959	0.5334	0.5361	0.116*	0.39
H20F	0.3276	0.6115	0.5133	0.116*	0.39
S2	0.19472 (9)	0.85254 (7)	0.32215 (5)	0.0510 (2)	
O4	0.3107 (3)	0.84047 (18)	0.22380 (12)	0.0666 (6)	
O5	0.0430 (3)	0.94944 (18)	0.31922 (14)	0.0715 (6)	
O6	0.8709 (2)	0.7722 (2)	0.55920 (14)	0.0725 (6)	
N3	0.1303 (3)	0.7306 (2)	0.37712 (18)	0.0558 (7)	
H3N	0.054 (4)	0.737 (3)	0.431 (2)	0.074 (10)*	
N4	0.5909 (3)	0.8385 (2)	0.63127 (17)	0.0464 (6)	
H4N	0.531 (3)	0.852 (2)	0.6812 (19)	0.048 (9)*	

### Atomic displacement parameters (Å^2^)

**Table:**

	*U*^11^	*U*^22^	*U*^33^	*U*^12^	*U*^13^	*U*^23^
C1	0.0546 (17)	0.0509 (17)	0.0336 (12)	−0.0121 (14)	−0.0065 (12)	−0.0141 (11)
C2	0.0633 (19)	0.0566 (18)	0.0351 (13)	−0.0090 (15)	−0.0115 (12)	−0.0208 (12)
C3	0.0663 (19)	0.0571 (19)	0.0360 (13)	−0.0076 (16)	−0.0162 (13)	−0.0170 (12)
C4	0.0681 (19)	0.0490 (16)	0.0412 (13)	−0.0069 (15)	−0.0184 (13)	−0.0159 (12)
C5	0.121 (3)	0.058 (2)	0.0444 (15)	−0.0006 (19)	−0.0344 (17)	−0.0244 (13)
C6	0.118 (3)	0.0550 (19)	0.0365 (14)	0.0003 (19)	−0.0320 (16)	−0.0167 (13)
C7	0.0550 (18)	0.0552 (18)	0.0432 (15)	−0.0082 (15)	−0.0049 (13)	−0.0207 (13)
C8	0.085 (2)	0.060 (2)	0.0606 (17)	−0.0049 (18)	−0.0142 (17)	−0.0184 (15)
C9	0.124 (3)	0.106 (3)	0.073 (2)	−0.041 (3)	0.021 (2)	−0.040 (2)
C10	0.134 (4)	0.087 (3)	0.138 (3)	−0.053 (3)	0.030 (3)	−0.034 (2)
S1	0.0857 (6)	0.0535 (5)	0.0532 (4)	−0.0020 (4)	−0.0284 (4)	−0.0203 (3)
O1	0.148 (2)	0.0633 (14)	0.0648 (12)	0.0019 (14)	−0.0616 (14)	−0.0176 (10)
O2	0.0702 (14)	0.0692 (14)	0.0750 (12)	0.0001 (11)	−0.0216 (11)	−0.0347 (10)
O3	0.0948 (16)	0.0751 (14)	0.0430 (11)	0.0051 (12)	−0.0103 (10)	−0.0294 (9)
N1	0.093 (2)	0.0600 (17)	0.0555 (16)	−0.0215 (15)	−0.0127 (16)	−0.0210 (13)
N2	0.0678 (16)	0.0517 (15)	0.0290 (11)	−0.0086 (12)	−0.0089 (11)	−0.0119 (10)
C11	0.0441 (16)	0.0447 (15)	0.0336 (12)	−0.0111 (13)	−0.0067 (11)	−0.0144 (10)
C12	0.0491 (17)	0.0536 (17)	0.0358 (13)	−0.0067 (14)	−0.0049 (12)	−0.0194 (11)
C13	0.0398 (15)	0.0555 (17)	0.0418 (13)	−0.0039 (13)	−0.0085 (12)	−0.0184 (12)
C14	0.0486 (17)	0.0481 (16)	0.0323 (12)	−0.0096 (13)	−0.0108 (11)	−0.0110 (11)
C15	0.0506 (17)	0.075 (2)	0.0350 (13)	−0.0133 (15)	−0.0029 (12)	−0.0230 (12)
C16	0.0415 (16)	0.080 (2)	0.0440 (14)	−0.0141 (15)	−0.0045 (12)	−0.0249 (13)
C17	0.0523 (18)	0.0467 (16)	0.0437 (14)	−0.0129 (14)	−0.0150 (13)	−0.0088 (12)
C18	0.0623 (19)	0.068 (2)	0.0596 (16)	−0.0101 (16)	−0.0260 (15)	−0.0193 (14)
C19	0.095 (3)	0.063 (2)	0.115 (3)	−0.013 (2)	−0.005 (2)	−0.034 (2)
C20A	0.187 (8)	0.071 (4)	0.080 (4)	−0.046 (5)	−0.037 (4)	−0.011 (3)
C20B	0.107 (8)	0.092 (8)	0.089 (7)	−0.002 (6)	−0.035 (6)	−0.030 (6)
S2	0.0567 (5)	0.0621 (5)	0.0360 (3)	−0.0101 (4)	−0.0169 (3)	−0.0136 (3)
O4	0.0735 (14)	0.1001 (16)	0.0299 (9)	−0.0264 (12)	−0.0085 (9)	−0.0191 (9)
O5	0.0702 (14)	0.0710 (14)	0.0708 (12)	0.0101 (12)	−0.0408 (11)	−0.0199 (10)
O6	0.0473 (12)	0.1133 (18)	0.0551 (11)	−0.0118 (12)	−0.0038 (10)	−0.0331 (11)
N3	0.0559 (16)	0.0708 (18)	0.0482 (13)	−0.0185 (14)	−0.0073 (12)	−0.0253 (12)
N4	0.0441 (15)	0.0603 (15)	0.0364 (12)	−0.0083 (12)	−0.0064 (11)	−0.0206 (11)

### Geometric parameters (Å, °)

**Table:**

C1—C6	1.381 (3)	C12—H12	0.9300
C1—C2	1.388 (3)	C13—C14	1.383 (3)
C1—N2	1.399 (3)	C13—H13	0.9300
C2—C3	1.363 (3)	C14—C15	1.377 (3)
C2—H2	0.9300	C14—S2	1.756 (2)
C3—C4	1.373 (3)	C15—C16	1.381 (3)
C3—H3	0.9300	C15—H15	0.9300
C4—C5	1.388 (3)	C16—H16	0.9300
C4—S1	1.756 (3)	C17—O6	1.208 (3)
C5—C6	1.351 (4)	C17—N4	1.351 (3)
C5—H5	0.9300	C17—C18	1.492 (3)
C6—H6	0.9300	C18—H18A	0.9600
C7—O3	1.215 (3)	C18—H18B	0.9600
C7—N2	1.343 (3)	C18—H18C	0.9600
C7—C8	1.491 (4)	C18—H18D	0.9600
C8—H8A	0.9600	C18—H18E	0.9600
C8—H8B	0.9600	C18—H18F	0.9600
C8—H8C	0.9600	C19—C20B	1.343 (8)
C8—H8D	0.9600	C19—N3	1.451 (4)
C8—H8E	0.9600	C19—C20A	1.471 (6)
C8—H8F	0.9600	C19—H19A	0.9700
C9—N1	1.463 (4)	C19—H19B	0.9700
C9—C10	1.484 (5)	C19—H19C	0.9700
C9—H9A	0.9700	C19—H19D	0.9700
C9—H9B	0.9700	C20A—H19C	1.2249
C10—H10A	0.9600	C20A—H20A	0.9600
C10—H10B	0.9600	C20A—H20B	0.9600
C10—H10C	0.9600	C20A—H20C	0.9600
S1—O1	1.4180 (19)	C20B—H20D	0.9600
S1—O2	1.431 (2)	C20B—H20E	0.9600
S1—N1	1.605 (3)	C20B—H20F	0.9600
N1—H1N	0.79 (3)	S2—O5	1.424 (2)
N2—H2N	0.80 (2)	S2—O4	1.4298 (17)
C11—C16	1.380 (3)	S2—N3	1.604 (2)
C11—C12	1.381 (3)	N3—H3N	0.83 (3)
C11—N4	1.407 (3)	N4—H4N	0.75 (2)
C12—C13	1.364 (3)		
			
C6—C1—C2	118.5 (2)	C13—C14—S2	119.3 (2)
C6—C1—N2	117.6 (2)	C14—C15—C16	120.1 (2)
C2—C1—N2	123.8 (2)	C14—C15—H15	120.0
C3—C2—C1	119.9 (2)	C16—C15—H15	120.0
C3—C2—H2	120.0	C11—C16—C15	119.9 (2)
C1—C2—H2	120.0	C11—C16—H16	120.1
C2—C3—C4	121.0 (2)	C15—C16—H16	120.1
C2—C3—H3	119.5	O6—C17—N4	123.2 (2)
C4—C3—H3	119.5	O6—C17—C18	121.6 (3)
C3—C4—C5	119.1 (2)	N4—C17—C18	115.2 (2)
C3—C4—S1	120.80 (19)	C17—C18—H18A	109.5
C5—C4—S1	120.1 (2)	C17—C18—H18B	109.5
C6—C5—C4	119.9 (2)	H18A—C18—H18B	109.5
C6—C5—H5	120.0	C17—C18—H18C	109.5
C4—C5—H5	120.0	H18A—C18—H18C	109.5
C5—C6—C1	121.5 (2)	H18B—C18—H18C	109.5
C5—C6—H6	119.3	C17—C18—H18D	109.5
C1—C6—H6	119.3	H18A—C18—H18D	141.1
O3—C7—N2	123.0 (3)	H18B—C18—H18D	56.3
O3—C7—C8	121.9 (2)	H18C—C18—H18D	56.3
N2—C7—C8	115.1 (2)	C17—C18—H18E	109.5
C7—C8—H8A	109.5	H18A—C18—H18E	56.3
C7—C8—H8B	109.5	H18B—C18—H18E	141.1
H8A—C8—H8B	109.5	H18C—C18—H18E	56.3
C7—C8—H8C	109.5	H18D—C18—H18E	109.5
H8A—C8—H8C	109.5	C17—C18—H18F	109.5
H8B—C8—H8C	109.5	H18A—C18—H18F	56.3
C7—C8—H8D	109.5	H18B—C18—H18F	56.3
H8A—C8—H8D	141.1	H18C—C18—H18F	141.1
H8B—C8—H8D	56.3	H18D—C18—H18F	109.5
H8C—C8—H8D	56.3	H18E—C18—H18F	109.5
C7—C8—H8E	109.5	C20B—C19—N3	117.2 (5)
H8A—C8—H8E	56.3	C20B—C19—C20A	54.7 (4)
H8B—C8—H8E	141.1	N3—C19—C20A	117.3 (4)
H8C—C8—H8E	56.3	C20B—C19—H19A	55.6
H8D—C8—H8E	109.5	N3—C19—H19A	108.0
C7—C8—H8F	109.5	C20A—C19—H19A	108.0
H8A—C8—H8F	56.3	C20B—C19—H19B	134.6
H8B—C8—H8F	56.3	N3—C19—H19B	108.0
H8C—C8—H8F	141.1	C20A—C19—H19B	108.0
H8D—C8—H8F	109.5	H19A—C19—H19B	107.2
H8E—C8—H8F	109.5	C20B—C19—H19C	108.1
N1—C9—C10	110.3 (3)	N3—C19—H19C	108.0
N1—C9—H9A	109.6	C20A—C19—H19C	55.8
C10—C9—H9A	109.6	H19A—C19—H19C	143.9
N1—C9—H9B	109.6	H19B—C19—H19C	58.7
C10—C9—H9B	109.6	C20B—C19—H19D	108.4
H9A—C9—H9B	108.1	N3—C19—H19D	107.5
C9—C10—H10A	109.5	C20A—C19—H19D	135.0
C9—C10—H10B	109.5	H19A—C19—H19D	59.1
H10A—C10—H10B	109.5	H19B—C19—H19D	50.6
C9—C10—H10C	109.5	H19C—C19—H19D	107.2
H10A—C10—H10C	109.5	C19—C20A—H19C	40.9
H10B—C10—H10C	109.5	C19—C20A—H20A	109.5
O1—S1—O2	119.76 (14)	H19C—C20A—H20A	113.9
O1—S1—N1	107.58 (15)	C19—C20A—H20B	109.5
O2—S1—N1	104.88 (13)	H19C—C20A—H20B	70.0
O1—S1—C4	107.82 (12)	C19—C20A—H20C	109.5
O2—S1—C4	108.01 (12)	H19C—C20A—H20C	133.9
N1—S1—C4	108.34 (14)	C19—C20B—H20D	109.5
C9—N1—S1	121.8 (2)	C19—C20B—H20E	109.5
C9—N1—H1N	116 (3)	H20D—C20B—H20E	109.5
S1—N1—H1N	110 (2)	C19—C20B—H20F	109.5
C7—N2—C1	129.0 (2)	H20D—C20B—H20F	109.5
C7—N2—H2N	117.9 (19)	H20E—C20B—H20F	109.5
C1—N2—H2N	113.0 (19)	O5—S2—O4	119.04 (11)
C16—C11—C12	119.4 (2)	O5—S2—N3	106.63 (14)
C16—C11—N4	123.5 (2)	O4—S2—N3	107.26 (13)
C12—C11—N4	117.0 (2)	O5—S2—C14	109.07 (12)
C13—C12—C11	120.7 (2)	O4—S2—C14	107.35 (12)
C13—C12—H12	119.6	N3—S2—C14	106.90 (11)
C11—C12—H12	119.6	C19—N3—S2	119.3 (2)
C12—C13—C14	119.9 (2)	C19—N3—H3N	115 (2)
C12—C13—H13	120.1	S2—N3—H3N	110 (2)
C14—C13—H13	120.1	C17—N4—C11	128.9 (2)
C15—C14—C13	119.8 (2)	C17—N4—H4N	117 (2)
C15—C14—S2	120.78 (18)	C11—N4—H4N	114 (2)
			
C6—C1—C2—C3	2.8 (4)	N4—C11—C12—C13	−177.1 (2)
N2—C1—C2—C3	−178.5 (2)	C11—C12—C13—C14	−0.8 (4)
C1—C2—C3—C4	−0.8 (4)	C12—C13—C14—C15	−1.9 (4)
C2—C3—C4—C5	−1.3 (4)	C12—C13—C14—S2	173.90 (19)
C2—C3—C4—S1	177.6 (2)	C13—C14—C15—C16	1.7 (4)
C3—C4—C5—C6	1.5 (5)	S2—C14—C15—C16	−174.1 (2)
S1—C4—C5—C6	−177.5 (3)	C12—C11—C16—C15	−3.9 (4)
C4—C5—C6—C1	0.6 (5)	N4—C11—C16—C15	176.9 (2)
C2—C1—C6—C5	−2.7 (5)	C14—C15—C16—C11	1.3 (4)
N2—C1—C6—C5	178.5 (3)	C15—C14—S2—O5	−141.0 (2)
C3—C4—S1—O1	7.2 (3)	C13—C14—S2—O5	43.2 (2)
C5—C4—S1—O1	−173.8 (3)	C15—C14—S2—O4	−10.8 (3)
C3—C4—S1—O2	138.0 (2)	C13—C14—S2—O4	173.46 (19)
C5—C4—S1—O2	−43.1 (3)	C15—C14—S2—N3	104.0 (2)
C3—C4—S1—N1	−108.9 (3)	C13—C14—S2—N3	−71.7 (2)
C5—C4—S1—N1	70.0 (3)	C20B—C19—N3—S2	102.6 (6)
C10—C9—N1—S1	178.0 (3)	C20A—C19—N3—S2	164.8 (4)
O1—S1—N1—C9	−49.8 (3)	O5—S2—N3—C19	179.1 (2)
O2—S1—N1—C9	−178.3 (3)	O4—S2—N3—C19	50.5 (3)
C4—S1—N1—C9	66.5 (3)	C14—S2—N3—C19	−64.4 (3)
O3—C7—N2—C1	−2.7 (5)	O6—C17—N4—C11	2.5 (4)
C8—C7—N2—C1	177.0 (3)	C18—C17—N4—C11	−177.5 (2)
C6—C1—N2—C7	−166.0 (3)	C16—C11—N4—C17	−6.6 (4)
C2—C1—N2—C7	15.2 (4)	C12—C11—N4—C17	174.2 (3)
C16—C11—C12—C13	3.7 (4)		

### Hydrogen-bond geometry (Å, °)

**Table:**

*D*—H···*A*	*D*—H	H···*A*	*D*···*A*	*D*—H···*A*
N1—H1N···O2^i^	0.79 (3)	2.13 (3)	2.914 (3)	173 (3)
N2—H2N···O4^ii^	0.80 (2)	2.21 (2)	3.006 (3)	169 (3)
N3—H3N···O6^iii^	0.83 (3)	2.03 (3)	2.854 (3)	173 (3)
N4—H4N···O3^iv^	0.75 (2)	2.21 (2)	2.960 (3)	174 (3)

Symmetry codes: (i) −*x*, −*y*+1, −*z*+2; (ii) *x*, *y*−1, *z*+1; (iii) *x*−1, *y*, *z*; (iv) *x*, *y*+1, *z*.
